# Auto Diagnostics of Lung Nodules Using Minimal Characteristics Extraction Technique

**DOI:** 10.3390/diagnostics6010013

**Published:** 2016-03-04

**Authors:** Diego M. Peña, Shouhua Luo, Abdeldime M. S. Abdelgader

**Affiliations:** 1 Department of Digital Image Processing, Faculty of Biomedical Engineering, Southeast University, Nanjing 210096, China; 220123712@seu.edu.cn (D.M.P.); Abdeldime@hotmail.com (A.M.S.A.); 2 Department of Electrical and Computer Engineering, Karary University, Khartoum 12304, Sudan

**Keywords:** lung nodule, segmentation, image processing, nodule characteristics

## Abstract

Computer-aided detection (CAD) systems provide useful tools and an advantageous process to physicians aiming to detect lung nodules. This paper develops a method composed of four processes for lung nodule detection. The first step employs image acquisition and pre-processing techniques to isolate the lungs from the rest of the body. The second stage involves the segmentation process using a 2D algorithm to affect every layer of a scan eliminating non-informative structures inside the lungs, and a 3D blob algorithm associated with a connectivity algorithm to select possible nodule shape candidates. The combinations of these algorithms efficiently eliminate the high rates of false positives. The third process extracts eight minimal representative characteristics of the possible candidates. The final step utilizes a support vector machine for classifying the possible candidates into nodules and non-nodules depending on their features. As the objective is to find nodules bigger than 4mm, the proposed approach demonstrated quite encouraging results. Among 65 computer tomography (CT) scans, 94.23% of sensitivity and 84.75% in specificity were obtained. The accuracy of these two results was 89.19% taking into consideration that 45 scans were used for testing and 20 for training. The rate of false positives was 0.2 per scan.

## 1. Introduction

Lung cancer is one of the most lethal cancers around the world, presenting the highest incidence rates 13% over all different cases of cancer diagnosed. In Asia, the Democratic Republic of Korea and China have the biggest incidence rate of this cancer [1]. According to the IARC (International Agency for Research on Cancer) the main cause with 75% of attribution of lung cancer disease is tobacco. A British study shows that the risk for a current smoker is 15 times higher than a never smoker [2]. Another important cause reported in Canada by McGill University Health Center shows that the air pollution increases the risk of different diseases [3]. This work seeks an alternative that could help to detect lung cancer in a short period of time.

At present, radiography and computer tomography (CT) have been improved, becoming potential methods of non-invasive imaging techniques. These improvements allow the observation of a detailed image. It increases the understanding of the internal anatomy in any part of the body [4]. One major example is the computer-aided detection (CAD) systems which offer an enormous contribution to assist physicians in the search and identification of nodules. The central benefit of this system is a faster diagnosis [5]. In addition, previous experience shows remarkable performance improvements of specialist physicians when they are aided by these systems. Identifying lung nodules in early stages has been the main intention for CAD researchers, it proves that the use of this method could lead to a cure or longer survival of people suffering of this decease [6]. Over the last 15 years, there have been studies dealing with the same issue, specifically with the idea to find a method for automatic detection of lung nodules [7].

Lung nodule detection has been proposed in many research studies and with that a lot of methodologies of identification were acknowledged. This works’ methodologies can be classified by three important stages: firstly lung extraction, secondly internal segmentation and thirdly False Positive (FPs) reduction. These three stages allow the works to complete a nodule detection following different algorithms and methods.

Lung extraction represents the primary step of the nodule detection. However, the extraction is not the focus point; the lungs reconstruction and threshold selection are the key points in this stage, because there are always some nodules linked to the pleura that cause difficulties. Some works [8,9,10,11] used different algorithms for lung reconstruction, among those options some use filling holes algorithm, morphology to define the lungs perimeter or connectivity to reconstruct the lungs volume and verify it as a solid one. Also there are other studies [10,12] with the main task in the threshold decision, the option of this methodology is an adaptive or a minimum error threshold which decides a parameter for lung extraction. The combination of both methods to develop a robust lung extraction represents a significant step for our algorithm of lung nodule detection.

The internal segmentation is divided into 2D and 3D methods which represent an initial identification of nodules. The 2D methods concenter mostly in the largest image to obtain characteristics and initialize a differentiation [13], aiming for the reduction of computational complexity. A vast amount of 3D algorithms were proposed targeting spherical volumes mostly because of the similarity to nodules [8,10]. There were also other algorithms focusing in the width of the structures in order to differentiate between tubes and round objects [9,11,12,13,14]. 3D methods require greater processing time and find a noticeable amount of FPs per scan leading towards more demanding classification characteristics.

The third category the reduction of FPs was broadly proposed by many researchers. Most of them use a Support Vector Machine (SVM) classification method [10,11,15], which represents one of the leading types of classification in this matter, owing to its ability to perform a non-linear differentiation when unbalanced data is present [16].

The primary objective of this paper is to achieve a precocious analysis of lung nodules segmentation and recognition between different lungs’ components using computer tomography images. This work uses minimal number of characteristics to provide a better description of the nodules and performs a remarkable classification by simplifying the procedure and methodology. The aim is to let the specialist physicians know about the existence, anatomy, and physical properties of the nodule candidates, consequently proper steps can be determined according to the specialist examination. To achieve lung nodule detection, we combined three different segmentation algorithms, which are a 2D threshold, a 3D blob detector, and a connectivity analysis. These coupling algorithms are proposed to impact each layer of the scan and detach the structures with features that are similar to nodules assuring a minimal false positive rate. Above all, this paper proposes a minimal feature extraction of possible nodule candidates, using eight vital describing characteristics from the possible nodules aiming to differentiate the candidates’ physical properties and texture peculiarities. Furthermore, a SVM classification method to select the real nodule structures using the least representative features that were obtained in the last step is employed.

The arrangement of this paper is divided as follows: Section 2 presents the materials and methods in four parts: first, the image acquisition and lung extraction; second, the segmentation techniques that were used to detect all possible candidates; third, the extraction of shape and texture features of the candidates; and lastly, the classification algorithm. In Section 3, we analyze the results of the algorithm obtained and we compare those results with other works that aim a similar solution for discussion. Finally, in Section 4, we present the principal remarks of this work in the conclusion.

## 2. Materials and Methods

**Figure 1:**
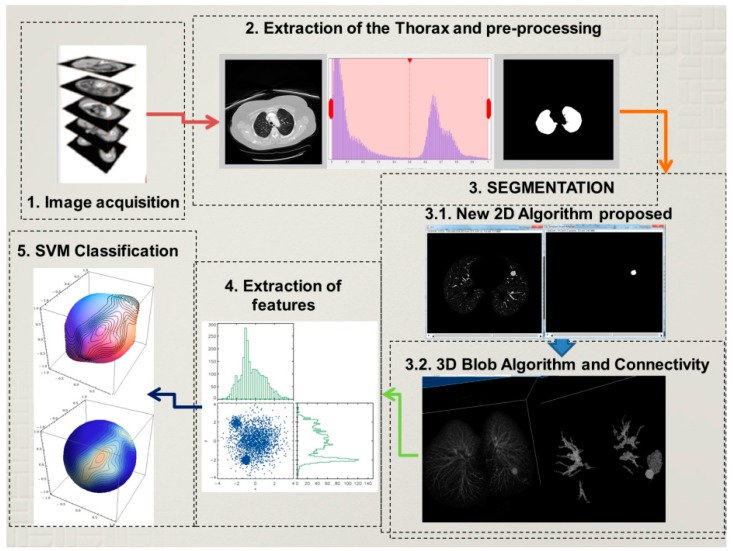
Lung Nodule Detection Process.

The detection of lung nodules proposed in this paper follows four principal steps that will be explained below. Firstly, the acquisition of the computer tomography images of the chest. Then a reduction of fat, muscles, and bones follows which involves image processing techniques to extract the lungs, and in some cases a reconstruction of the lung parenchyma is carried out. This reconstruction is necessary when the nodules are connected to the pleura, because while using the normal procedure these nodules could be erased. Secondly, a 2D algorithm of region and intensity analysis is applied in every layer of the stalk of images to achieve a satisfactory result in the lung structure segmentation. The purpose is to eliminate a considerable amount of structures that are irrelevant and unnoticeable such as small vessels, bronchi, *etc.* Subsequently, a 3D blob algorithm and a connectivity algorithm are used to order, select and label remaining structures that at first glance could represent lung nodules. Thirdly, an extraction of characteristics helps to detect geometric and physical parameters in the structures that could describe them with a higher precision to obtain the nodules with the least number of characteristics. Finally, the structures are classified using a support vector machine (SVM) which is a learning algorithm machine that will use the characteristics obtained before to classify nodules and non-nodules. The proposed methodology is described using a block diagram in Figure 1.

### 2.1. Acquisition and Extraction of the Lungs

The initial data used to pursue this work are chest images. In these images, there are other components that are not useful for the corresponding research such as organs, tissue, bones, and objects like the bed of the CT machine. Therefore, the procedure starts with the extraction of external structures from the chest images to obtain the lungs. This step is essential for the next stages for two main reasons: to concentrate resources and time on the region of interest and not to misplace nodules that are linked to the pleura. The last ones create a big problem in nodule segmentation and recognition. As it was explained, this fundamental step prepares the stalk images of the chest to focus only on the lungs for the following processes.

To cover this step, there were some image processing methods that were carried out in the following order. The process starts with obtaining a histogram of the image. This was performed for two main reasons: first to reason is to normalize the stalks and the second one to have an idea of the intensity levels. As it is known from the other research [14] the intensity levels of these images show two well-defined picks: one represents the internal structures of the lungs which are the intention of the research and the other represents fat, muscles and harder structures of the images. The core idea is to work with the histogram and use a threshold point. However, it is not the most recommended action because there could be some nodules that are linked to the pleura. In these cases, the histogram method would erase the nodules. As a result, there will be a loss of the region of interest (ROI) through the next steps. For this reason, we follow a minimum error thresholding algorithm because this treats every case as a new one and according to each case of dataset and it selects a new threshold value [17].

### 2.2. Segmentation of the Lung Structures

The segmentation of lung structures is a key direction of this work, because it aims to search, separate, and differentiate between all the structures inside the lungs. This stage is performed in two steps: the first step works with a 2D algorithm using image processing techniques and intensity analysis to remove less dense parenchyma tissue and non-informative data structures from the slices. The second step includes a 3D blob algorithm and connectivity algorithm to identify, separate all the structures that remain, and eliminate a notable amount of false positives.

#### 2.2.1. 2D Segmentation Algorithm

The method used to initiate the segmentation is focused on every layer of the stock of images. To accomplish this procedure, initially it is important to enhance the bright structures around the ROI, in this case the lungs. Then the algorithm recognizes every participant in the image and labels it as a single structure. The objective of this step is to extract the following features of every single structure: the number of pixels, area, and intensity. Then, a threshold process is used with this information. The threshold values of area and intensity help to eliminate some small structures that cannot be visible because of size or brightness. These elements are taken out as irrelevant pieces for this nodule detection.

**Figure 2:**
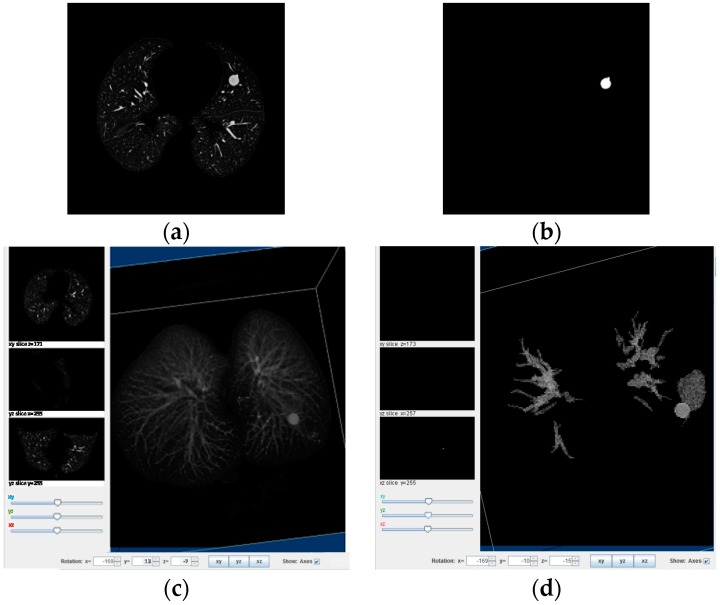
2D algorithm (**a**,**b**) 2D images of the procedure before and after; (**c**,**d**) 3D images of the procedure showing a proportional reduction of pieces.

As a matter of fact, it is difficult to choose a proper threshold for elimination of structures, for this reason there has to be two indicators that must agree to make this decision smoother and open to prospective candidates that could represent a nodule. The threshold values have been chosen according to two measurements; first a histogram which shows the intensity levels of the image, and the size of the smallest nodule that this paper is aimed to find. The nodule size limit used in this paper is 4 mm, because we build on the strength of the result obtained by [18] in which nodules smaller than 4 mm have a 0% malingnancy and the CT resolution for such small nodules can be treated as noise by the algorithm. The 2D Algorithm helps to identify relevant structures in every layer of the stock of images, as it can be seen in Figure 2a the lungs internal structures are shown. In the next Figure 2b the reduction of internal structures can be represented only by one important piece which in this case constitutes the nodule. The last Figure 2c,d show the 3D representation of the reduction of internal structures and the importance of this procedure for further analysis.

#### 2.2.2. 3D Blob Algorithm and Connectivity Analysis

For this step, the algorithm must start with the reconstruction of the 3D CT scan denoted as I(x,y,z) where now z represents the slices of every image and x,y each coordinate of every slice. After the 2D algorithm as shown in Figure 2d the internal structures have been considerably reduced. For this reason, a 3D lung reconstruction will be helpful to visualize the certain relationships, such as connectivity or interaction between slices.

This connectivity analysis searches for all the structures and components that are connected in the 3D image. A suitable connectivity could be a 26 connected neighbors to unite the corresponding participants. These 26 adjoined voxels are being analyzed, starting from the central voxels which have neighbors that share edge faces and adjacent corners with the rest of the voxels one by one connecting and forming a structural neighborhood.

The 3D blob detector has been used and analyzed in several research publications [8,12,19,20]. The main drawback of this method is the high amount of false positive detections. This algorithm especially confuses some middle points where the ramifications of the vessels spread and wider vessel branches [19]. However, lung nodules mostly appeared as blob brighter elements than the rest of the pulmonary background. The rest of the nodules have some irregular shape which logically increases the false positive apparition.

### 2.3. Extraction of Features

**Figure 3:**
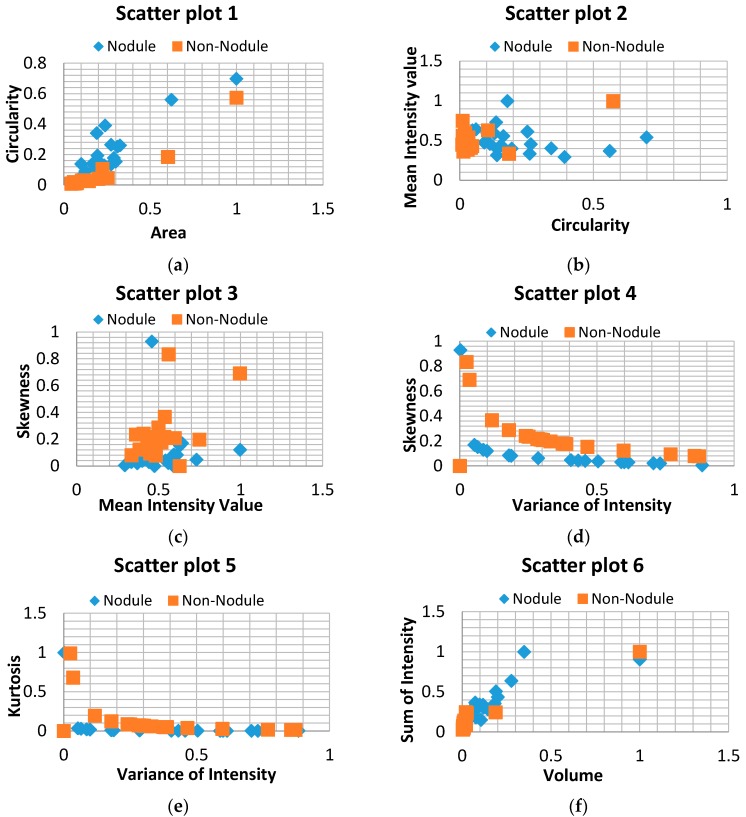
Scatter plots showing the relationship of features between (**a**) Area and Circularity; (**b**) Circularity and Mean intensity value; (**c**) Mean intensity value and Skewness; (**d**) Variance of Intensity and Skewness; (**e**) Variance of intensity and Kurtosis; (**f**) Volume and Sum of Intensity.

After the segmentation process, further examination is required to be conducted in the remaining structures. This analysis is carried out by feature differentiation. The procedure aims to perform a selection of features that could describe the main attributes of lung nodules compared with non-nodules. There are two main sources for feature extraction that have been used, one source is through geometrical measurements (area, diameter, circularity, effective radius, discircularity, elongation, volume, compactness, spherical disproportion, spherical density), and the other by histogram measurements (mean intensity value, variance of intensity, sum of intensity, skewness, kurtosis). From these 16 features only the most representative need to be selected using a correlation analysis between every two characteristics. The eight core features of this work have been selected by this analysis as can be seen in Figure 3 [21,22]. The result brought the following marker features: as geometrical measurements (area, circularity, volume), and for histogram measurements (mean intensity value, variance of intensity, skewness, kurtosis, sum of intensity). The detailed equations and description of the characteristics that have been selected are explained in Table 1.

### 2.4. Structural Classification

The Support Vector Machine (SVM) has strong regularization properties with theoretical foundations based on Vapnik-Chervonenkis theory aiming to classify information [7]. This algorithm is very sustainable for unbalanced data which is crucial reason to be used in this work; moreover, SVM has a tremendous advantage classifying non-linear cases which is another important characteristic of nodules. However, this algorithm needs to perform some steps as training and classification tool before being used. The information for both training as well as for classification is different; therefore, training data and new data (classification data) need to be normalized together and then split to perform the training or classification respectably.

The library used to fulfill this purpose is LIBSVM [16], this library is a variety of kernels and ways of classification, all depending on the data that is being used and on the number of dimensions a classification must require. There are four types of kernels that can be used in an SVM classification; in this work Radial Basis Function (RBF) is used. The other parameter to be selected to work with this kernel is elipson which represents the loss of the Support Vector Regression (SVR). This elipson parameter is presented when the RBF kernel is acting using a SVR model which is the case of this work. Moreover, this library offers a trained validation of the classifiers and automatic selection of the C and γ parameters that will be set automatically in the RBF kernel.

## 3. Results and Discussion

The images used in this paper have been obtained from The Cancer Imaging Archive (TCIA) [23], the first part involved a database accessible to public download focusing on lung cancer [24,25]. The second is a database representing the contest of lung nodule identification supported by the international society for optics and photonics (SPIE), this database was developed to encourage CAD systems to perform a more exquisite lung nodule detection using special cases [26,27]. The selection of data used in this work is a mixture of both databases TCIA and SPIE competition. All these data were evaluated by chest radiologists who detected the exact locations of nodules and basic measurements of convexity and entropy of them. The validation of this work efficiency was done by a cross reference technique with the expert’s evaluation in order to determine the accuracy of the proposed work.

A total of 131 images were found in the cancer imaging archive, from which 61 CT images belong to the TCIA focus on lung cancer and 70 CT images correspond to the SPIE 2015 competition of lung nodule detection. The first database contributes with 30 random scans; these scans are TCIA cases within R_004 to R_274. As for the second database, 35 scans were collected from LUNGx-CT001 to LUNGx-CT070. In total 65 scans were used in this work, from which at final stage 20 scans are part of a training set, and the rest belongs to the evaluation to measure the classification sensitivity. All the scans showed a certified proof of their nodule location and their basic parameters were confirmed by specialists.

The 65 scans of this research have different cases containing one or two nodules. The only limitation for this work, as mention before, is the nodule size, which limits the work for nodules bigger than 4 mm, as for the rest nodules come in different shapes, intensities and locations. The characteristics of the scans will be described in the following lines: beginning with the number of slices of every stalk of images that varies from 70 to 400, which represents an average value of 153 slices per scan. Other characteristic is the slice thickness which controls the pixels size range that fluctuates from 1.40 to 3 mm and finally the resolution of scan which is 512 × 512 pixel. The complete selected dataset had different kinds of lung nodules which consisted of isolated nodules, juxtapleural nodules, vascular nodules, and nodules with low contrast intensity. Additionally, it is important to remark that those nodules represent all stages of lung cancer and every phase of the disease.

### 3.1. Detection Procedure

**Figure 4:**
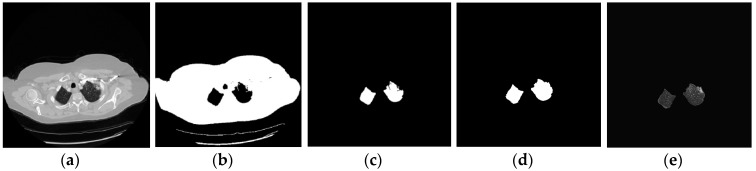
This is one successful example of lung extraction while the nodule is linked to the pleura (**a**) CT image showing all the structures; (**b**) Binary image after the threshold; (**c**) Lung selection for extraction with area loos; (**d**) Lung completed after the application hole-filling algorithm; (**e**) The lung extracted showing the internal structures.

In this section, we will describe the different steps by which this nodule segmentation research works. The first step starts with the adjustment of the image to show the lungs clearly and also to enhance some other objects in the process. After that, the minimum error thresholding algorithm separates two regions in order to avoid loss of lungs parts; however, there are always some cases that contain nodules too bright near the pleura or nodules bigger than the lungs that break one lung perimeter, as shown in Figure 4. For example, in Figure 4a it can be noticed that there is a small nodule near the pleura in the upper right side of the right lung. Normally, the nodule disappeared using the minimum error method, as can be seen in Figure 4b,c the nodule disappeared. For these cases, a hole-filling algorithm must be applied to prevent an imminent loss of vital information. After that step, in Figure 4d,e the result showed that the nodule is reconstructed and the data is placed again into the image for the next procedure.

**Figure 5:**
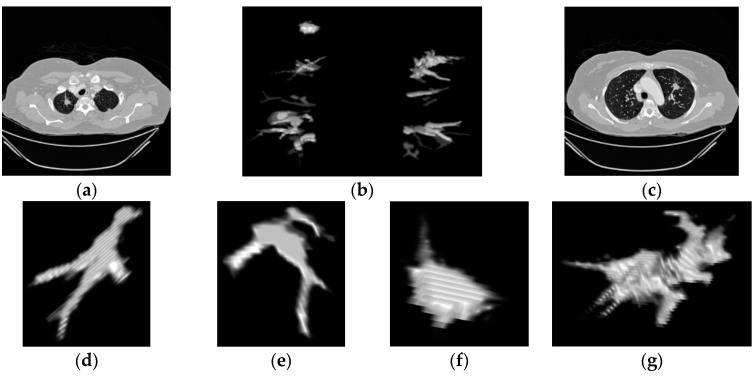
Precise Lung nodules detection (**a**) CT image containing the first nodule; (**b**) Lung 3D reconstruction; (**c**) CT image containing the second nodule; (**d**–**g**) all possible candidates before classification; (**d**,**e**) Non-nodules; (**f**,**g**) lung nodules determine after classification.

**Figure 6:**
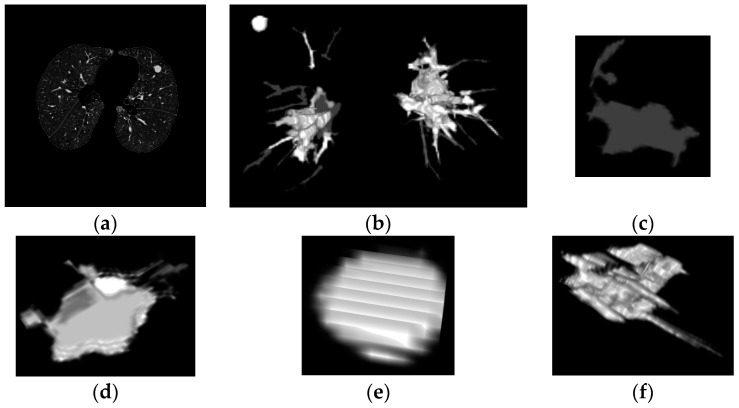
Lung nodule detection with false positives (**a**) CT image showing all the structures; (**b**) Lung 3D reconstruction; (**c**–**f**) all possible candidates before classification; (**c**,**d**) Non-nodules; (**e**) lung nodules determine after classification; (**f**) False positive presented in the detection.

After the lung extraction, the next process is the segmentation of nodules that first involves a 2D analysis, in every layer of the volume. This analysis mainly involves the intensity, the area and the representativeness to determine the parts that need to be enhanced inside the lungs. The parenchyma and structures below the threshold limits are normally deleted. As an example, In Figure 5a,c and Figure 6a the principal images of the CT scan can be seen showing the nodules that are found in the lungs. Then, in Figure 5b and Figure 6b the 3D reconstruction of the lungs after the 2D analysis is presented, as it can be observed, the blocks show all the significant reduction after that process.

Hereafter, two principal processes will act together: a 3D connectivity analysis and a blob algorithm. The 3D connectivity analysis created structures from the remaining pieces in every layer of the scan; this procedure identified 3D formations. The blob algorithm measured the volume’s sphericity to determine basic measurements of possible nodules. Then, all the structures are discriminated by sphericity and width to reduce quantity of candidates. Correspondingly, the structures that remain are shown in Figure 5 and Figure 6 representing possible nodule candidates.

The next step performs the characteristic extraction of the remaining structures. The matrix of characteristics is composed of the following descriptors: circularity, effective radius, elongation, compactness, mean intensity value, variance of intensity, skewness. These eight markers were selected from correlation diagrams applied between these characteristics. Figure 3 shows the correlation diagrams that represent the difference of nodules and non-nodules. Even if there is a small overlap between them, the trends of distribution show a reliable and clear classification. This step contributed to this work most of all, because it allows us to represent the difference using only eight simple characteristics. The quantity of features used in the classification represents a crucial decision of FPs reduction. In our case, the work accomplished the principal objectives by reducing the candidates feature descriptors to the minimum and allowing an easy discrimination of structures in the classification stage.

**Figure 7:**
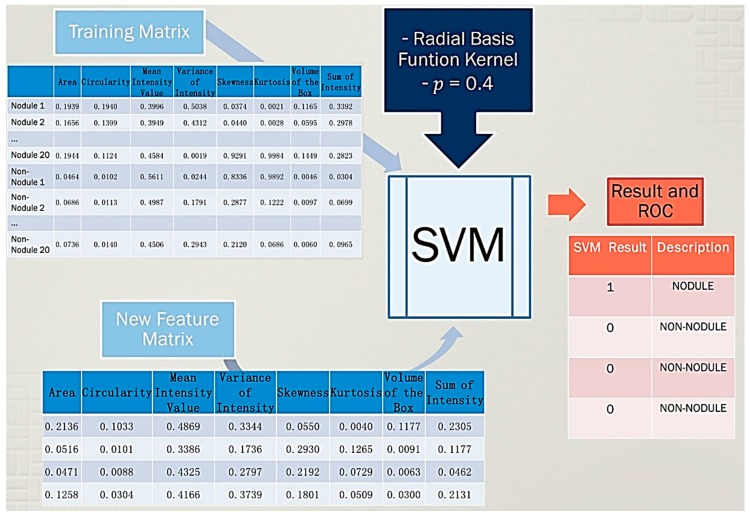
SVM classification procedure with real data of structural characteristics showing four possible candidates and the detection of one nodule and three non-nodule structures.

**Figure 8:**
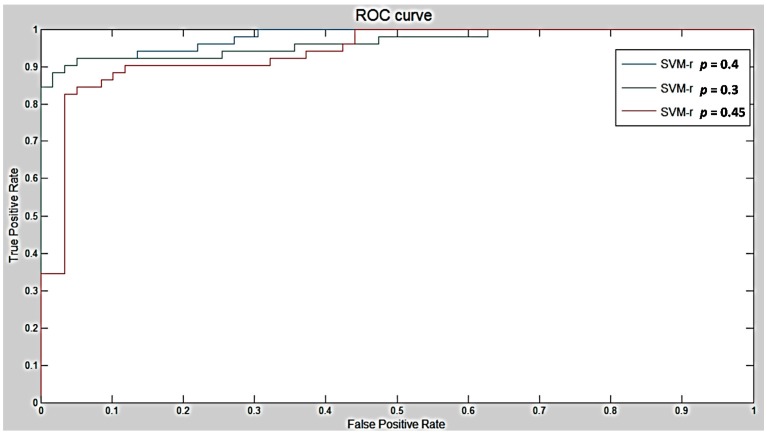
Receiver Operating Characteristic (ROC) Curves based on different epsilon index using the same radial in basis function kernel.

As it was explained in the last chapter, the SVM algorithm with a kernel in radial basis function was selected in this work, this kernel showed a better sensitivity in prediction compared to others [28]. An epsilon *p* = 0.4 was selected as it can be seen in Figure 7 and Figure 8. After that, five steps shown in Figure 7 were introduced to perform a classification using an SVM algorithm.

The first step for the SVM training involved the creation of a balanced matrix which in our case is a matrix that contains 20 nodules and 20 non-nodules structures with all characteristics as observed in Figure 7. The second step implicated joining the matrix of new structures obtained in the experiment with the balanced matrix together to perform a normalization of data. The third step is based on the use of the balanced matrix normalized to train the SVM algorithm. In the fifth step, the matrix of new structures normalized is classified using the SVM which as a result showed the nodules in the scan as the procedure in Figure 7.

In our cases of detection represented in Figure 5 and Figure 6 the first two structures are non-nodules and the remaining two images were selected as nodules resulting in the classification process. In the first case in Figure 5f,g both structures are selected as lung nodules, these images represent the nodules showed in Figure 5a,c. The result of this first detection is correct and the two nodules were detected with high accuracy. In the second case of study in Figure 6e,f both structures were selected as lung nodules in the classification, but only Figure 6e is a real nodule, the other structure represents a bifurcation of vessels which is one FP structure that was detected by the SVM as a lung nodule. The possibility for the last structure being a nodule is high because there are many irregular nodules that can look like that structure. For this reason in this case the false positive detection is understandable. This sub-section explains the procedures of this work including two real examples, one showing a successful detection and the other showing a detection of a false positive structure with one real nodule.

### 3.2. Evaluation of the Methodology

Considering the significance of the research, there were two evaluations that have been considered in this work. The first one consisted of an evaluation of cross validation on every nodule sample [29]. This method took two arbitrary elements from the sample for testing, and then the rest is left for training. This procedure assures that all possible samples have been tested, and also that all of them have been the part of the training method. This process offers a highly consistent validation.

The second one is used to evaluate the classification capability, which involved the detection of false positive rates per exam. This method enabled us to know the sensitivity level. The valuable measurements of this method are: TP which are the correct nodules being classified, TN the correct structures that were not correspondent nodules, FP and FN represent the erroneous classified as nodules and non-nodules. Having obtained those last parameters, the sensitivity can be defined as TP/(TP + FN), the specificity by TN/(TN + FP) and the accuracy by (TP + TN)/(TP + TN + FP + FN). These three results allow us to rank the methodology used and mostly the classification [28].

These two evaluations were used to test all the possible nodules as testing and training samples. Also, learn more details about the candidates and determine the representation for each candidate. An example of representation can be seen in Figure 5g and Figure 6f one is a real nodule and the other is a false positive acquired after the classification, the resemblance of the structures is striking not only because of the size but also due to the other features. Lung nodules cannot only be spherical, they can also be represented by disproportional structures that make the classification a challenging task. Trying to acknowledge all the possible irregular forms is a difficult task given the training method; therefore, this process of treating all the nodules as training subjects helped to recognize the best representatives from nodules and non-nodules for training. This process allowed us to pick the extreme cases of real nodules.

As it was explained in the last sub-section the features of the SVM algorithm used in this work are the kernel in radial basis function and epsilon which is represented as *p*. The last indicator *p* can slightly control the behavior of the system resulting in a change of sensitivity in the classification. In Figure 8, three surrounded values are used as training parameters to obtain the highest sensitivity among classifications. As Figure 8 shows, the optimized epsilon obtained in the classification is represented by *p* = 0.4. The optimized value brought the next results: for sensitivity 94.23%, specificity 84.75% because there were three nodules missing in the test and some non-nodules that appear to be nodules in the test classification, at last the accuracy of the classification of 89.19% according to the quantity of TP and TN found in the experiment.

Lastly, a chart was built to detail the most important works that had been cited in this research which used different techniques of segmentation and most importantly their performance results. In Table 2, there are many kinds of works starting from primary research performed by Lee *et al.* [30] or some recent works issued last year by Saien *et* *al*. [12]; moreover, there is some research done by teams using different techniques as by Choi *et al.* and Silva *et al.* [9,10,11,14].

In all of the cited documents, there were some problems regarding the consumption of FPs. The sensitivity rate obtained by this work is something between other proposed works because of the specialized database used for testing. This prototype has been tested with the use of the SPIE competition for nodule detection database. The database contains special examples of nodules which have not previously been detected by several CAD systems. A second index to compare is the specificity of 84.7% which represents a low appearance of FPs 0.2 per scan. This index expresses a clear reduction of false positive structures appearance as seen in Table 2. That means, the proposed prototype provides a trade-off between sensitivity and false positive rate.

## 4. Conclusions

In this work, a robust compilation of segmentation algorithms was developed to detect lung nodules using only eight characteristics of the classification. The content initially involved image acquisition and concluded at lung nodule extraction. The performance of the presented technique was evaluated as a reliable methodology for lung nodule detection. Simple algorithms were combined to perform this procedure with satisfactory results; these accessible implementations of algorithms have a fast processing response. In medical equipment the time of response is crucial, because inside the work environment lives of human beings are involved. Three remarkable attributes of this methodology are quick response, simplicity of the algorithms configuration, and merest features extraction. These attributes provide essential help to the specialist. In addition, this beneficial methodology brings confidence in the disease detection and early diagnosis which are the specialists’ biggest hopes for treatment and management. Moreover, this procedure can operate in a highly scalable manner and use minimum time focusing only in CT scan machines using a global DICOM format standard. The segmentation highly recommended the 2D algorithm along with the 3D blob algorithm and the connectivity analysis. These algorithms performed a fundamental recognition of structures in every layer of the scan. Furthermore, they detected and reduced false positive appearances inside the lungs which would provide plenty of processing time. The matrix of characteristics was analyzed by the representativeness among every possible combination of characteristics using correlation diagrams differentiating nodule and non-nodule structures. This process allowed us to discover the highest descriptors among all features which were eight at the moment of classification. The classification performed by the SVM algorithm proved to be essential in this methodology, not only because many papers mention this algorithm, but also because it is sensible to multiple characteristic classification and it can be adapted to unbalanced data. Lung nodules are always less than non-nodules before the classification; this peculiarity makes the training matrix selection arduous. The evaluation method applied to measure the performance shows the following results: a sensitivity of 94.23% owing to the loss of three in a pile of 52 nodules when the algorithm was being tested, as a second indicator a specificity of 84.75% because of the appearance of FPs and finally the accuracy of this work obtained 89.19% which determined this work as a reliable perspective method. The appearance of false positives in the detection generated a big loss in specificity; however, this paper is proposed to be used by an expert physician who can determine the nature of the real nodule and a false positive.

## Figures and Tables

**Table 1:** Selected Features.

Features	Equation	Description
Area	$d_{1}=Scalar\;number\;of\;pixels\;\in \;PN$	The actual number of pixels in the region.
Circularity	d2=d14×π×(Diameter of PN2)2	Quantity representing the degree to which a shape is compact.
Mean intensity value	$d_{3}=\sum_{\left(x,y,z\right)\epsilon PN}\frac{f\left(x\right)}{number\;of\;pixels\;\in PN}$	Mean value of the image.
Variance of intensity	$d_{4}=Var\left(\left\{f\left(x,y,z\right):\left(x,y,z\right)\in PN\right\}\right)$	Variance of the intensity.
Skewness	$d_{5}=\frac{\frac{1}{n}\;\sum_{i=1}^{n}{\left(x_{i}-\bar{x}\right)}^{3}}{{\left(\sqrt{\frac{1}{n}\sum_{i=1}^{n}{\left(x_{i}-\bar{x}\right)}^{2}}\right)}^{3}}$	Measure of the asymmetry of the data around the sample mean.
Kurtosis	$d_{6}=\frac{\frac{1}{n}\;\sum_{i=1}^{n}{\left(x_{i}-\bar{x}\right)}^{4}}{{\left(\frac{1}{n}\;\sum_{i=1}^{n}{\left(x_{i}-\bar{x}\right)}^{2}\right)}^{2}}$	Measure of how outlier-prone a distribution is. A normal value is 3.
Volume of the box	$d_{7}=L_{BB}\times W_{BB}\times H_{BB}$	Volume of the boundary box.
Sum of intensity	$d_{8}=\sum_{\left(x,y,z\right)\epsilon PN}f\left(x\right)$	Addition of the Intensities

PN: Represents the Possible Nodules to be measure.

**Table 2:** Performance comparison of the methodology proposed with other research papers.

Reference Number	Dataset	Sensitivity %	FPs/Scan
[8]	18 CT scans/4853 slices/222 Nodules	95.9	-
[12]	42 CT scans/7346 slices/124 Nodules	95.9	38.8
[30]	32 Scans/5721 slices	100	-
[31]	7Scans/2449 slices/24 Nodules	80	7.7
[9]	84 Scans/average of 310 slices/148 Nodules	97.5	6.76
[14]	43 Scans/average of 240 slices/151 Nodules	95.28	2.27
[10]	33 Scans/average of 240 slices/33 Nodules	84.84	0.42
[11]	29 Scans/4949 slices/48 Nodules	85.93	0.138
This work	45 Scans/average of 153 slices/52 Nodules	94.23	0.2

## Abbreviations

The following abbreviations are used in this manuscript:

CAD: Computer Aided Detection
IARC: International Agency for Research on Cancer
CT: Computer Tomography
PFs: False Positives
ROI: Region of Interest
RBF: Radial Basis Function
SVR: Support Vector Regression
TCIA: The Cancer Imaging Archive
SPIE: The international Society for optics and photonics
DICOM: Digital Imaging and Communication in Medicine
